# Hairy/Enhancer-of-Split MEGANE and Proneural MASH1 Factors Cooperate Synergistically in Midbrain GABAergic Neurogenesis

**DOI:** 10.1371/journal.pone.0127681

**Published:** 2015-05-20

**Authors:** Clara-Zoe Wende, Saida Zoubaa, Alexandra Blak, Diego Echevarria, Salvador Martinez, François Guillemot, Wolfgang Wurst, Jordi Guimera

**Affiliations:** 1 Institute of Developmental Genetics, Helmholtz Zentrum München, Deutsches Forschungszentrum für Gesundheit und Umwelt (GmbH), Neuherberg, Germany; 2 Department of Neuropathology, Regensburg University Hospital, Regensburg, Germany; 3 Experimental Embryology Laboratory, Instituto de Neurociencias, Universidad Miguel Hernández, Alicante, Spain; 4 Division of Molecular Neurobiology, MRC National Institute for Medical Research, London, United Kingdom; Universitat Pompeu Fabra, SPAIN

## Abstract

GABAergic neurons are the primary inhibitory cell type in the mature brain and their dysfunction is associated with important neurological conditions like schizophrenia and anxiety. We aimed to discover the underlying mechanisms for dorsal/ventral midbrain GABAergic neurogenesis. Previous work by us and others has provided crucial insights into the key function of *Mgn* and *Mash1* genes in determining GABAergic neurotransmitter fate. Induction of dorsal midbrain GABAergic neurons does not take place at any time during development in either of the single mutant mice. However, GABAergic neurons in the ventral midbrain remained unchanged. Thus, the similarities in MB-GABAergic phenotype observed in the *Mgn* and *Mash1* single mutants suggest the existence of other factors that take over the function of MGN and MASH1 in the ventral midbrain or the existence of different molecular mechanisms. We show that this process essentially depends on heterodimers and homodimers formed by MGN and MASH1 and deciphered the *in vivo* relevance of the interaction by phenotypic analysis of *Mgn/Mash1* double knockout and compound mice. Furthermore, the combination of gain- and loss-of-function experiments in the developing midbrain showed co-operative roles for *Mgn* and *Mash1* genes in determining GABAergic identity. Transcription factors belonging to the Enhancer-of-split-related and proneural families have long been believed to counterpart each other’s function. This work uncovers a synergistic cooperation between these two families, and provides a novel paradigm for how these two families cooperate for the acquisition of MB-GABAergic neuronal identity. Understanding their molecular mechanisms is essential for cell therapy strategies to amend *GABAergic* deficits.

## Introduction

During brain development, neuronal cell specification is a fundamental process leading to the generation of neuronal subtypes. The combination of genetic cascades initiated by different factors and mechanisms, determine the timing, number, and neuronal identity of the nascent neurons at precise locations (reviewed in [1]). Gamma-aminobutyric acid (GABA) is the primary inhibitory neurotransmitter in the mature central nervous system. GABAergic neurons (GABAn) comprise a highly diverse group of neurons with important functions [2]. Neurological conditions such as schizophrenia, bipolar disorder, epilepsy, anxiety, Huntington’s disease, chronic pain, and addiction are associated with a marked dysfunction of GABAergic inhibition. Midbrain (MB) GABAn comprise diverse interneurons, distributed over 3 major subdomains of the MB neuroepithelium and further assigned into 7 molecular subdomains (m1-m7): i) dorsal MB (dMB) GABAn, encompassing the neurons of the superior and inferior colliculus (SC and IC, respectively) in the dorsal alar plate (m1 domain) and the laterodorsal periaqueductal gray area located in the lateral alar plate (m2 domain); ii) ventrolateral MB (vlMB) GABAn, comprising the neurons of the ventral periaqueductal gray area and reticular formation nuclei, located in the ventrolateral alar plate (m3 domain), lateral basal plate (m4 domain) and intermediate basal plate (m5 domain); and iii) ventral-most MB (vmMB) GABAn, corresponding to the neurons of the ventral tegmental area and substantia nigra in the medial basal plate (m6 domain) and floor plate (m7 domain).

The fact that different subclasses of MB-GABAn are formed at different times and locations raises the possibility that they may be generated by distinct factors and/or mechanisms. A number of studies have indicated that the transcription factors of the basic helix-loop-helix (bHLH) family play a conserved role in neurogenesis in vertebrates and invertebrates. In particular, neurogenesis depends on the balance between the members of the hairy/Enhancer-of-split (*h/E(spl)*) and proneural gene families. The former are the direct effectors of NOTCH signaling, which maintain NOTCH active cells in a progenitor state through lateral inhibition, whereas the proneural genes typically promote neuronal identity [3,4]. These two major groups act in mutual repression to control the number of neurons and their identity along the three primary brain vesicles of the neural tube: forebrain, MB, and hindbrain.

While MB-GABAn have been the subject of recent research on their genetic endowments (reviewed in [5]), less is known about how the apparent similarity of gene function specifically operates during MB-GABAergic neurogenesis. The dorsal/ventral mechanisms by which these transcription factors regulate the induction of MB-GABAn during development remains poorly understood. To better understand the molecular mechanisms by which MB-GABAn acquire their specific identity *in vivo*, which are different from the mechanisms operating in other parts of the CNS, we identified and characterized a novel bHLH gene referred to as *Megane* (*Mgn*) [6] or *Helt*_Mouse Genome Informatics (MGI). Expression of *Mgn* mRNA shows a specific and dynamic pattern in the embryonic CNS, which is developmentally controlled in a tissue-specific manner [6,7,8]. Tissue distribution and the ontogenetic expression pattern of *Mgn* show a spatio-temporal correlation with that of GABAergic markers (*Gad65/67*). Early expression of *Mgn* at embryonic day (E) 9.5 corresponds with the onset of the first GABAn, and its expression is turned off in all GABAn precursors immediately after they became postmitotic. *Mgn* encodes a protein that shares structural homology within the bHLH-O region to the products of *Drosophila h/E(spl)* genes. Despite this structural homology, MGN displays critical divergences in several characteristic residues that distinguish it from other subfamilies related to the h/E(spl) family, including the mammalian HES, HEY, SHARP and DEC1 subfamilies. Therefore, MGN constitutes a new subclass of bHLH transcription factors related to *h/E(spl)* [6]. Promoter binding analysis performed *in vitro* suggest MGN homodimers act as a transcriptional repressor by binding to E-box class B and C1 sequences, which are typical recognition sequences for bHLH repressors, but with a different preference for the core sequence when compared with other bHLH-O repressors [7]. Whether MGN activates neurogenesis *in vivo* by binding to E-box class A (typical recognition sequence for activators) or represses repressors of neurogenesis in either homodimer or heterodimer condition needs to be formally probed. From the functional point of view, MGN is associated with early steps in the specification of GABAergic lineages in the CNS rather than their maturation and functional maintenance and plays a key role as a primary selector of GABAergic neuronal identity in the MB [9,10].

The proneural bHLH gene *Mash1* (*Ascl1*_MGI) has been found to be necessary for MB-GABAergic neurogenesis [11]. Whereas *Mgn* is the earliest GABAergic marker expressed specifically in mitotic GABAn precursors, the expression of *Mash1* is maintained in many postmitotic cells of the basal and alar MB neuroepithelium. In contrast to *Mgn*, *Mash1* is also found in neurons that release neurotransmitters other than GABA, including noradrenergic, acetyl cholinergic, serotonergic, and glutamatergic lineages, as well as in oligodendrocytes and astrocytes (reviewed in [12]). MGN and MASH1 are critical intrinsic factors required for the specification of the dMB-GABAn, because these neurons fail to develop their GABAergic identity in *Mgn* or *Mash1* single mutants. Both mutants showed complete depletion of GABAn in the SC (m1–m2 domain). However, GABAn in the vlMB (m3–m5 domains) remained unchanged at E15.5. Induction of dMB GABAn does not take place at any time during development in either of the mutant mice, but the morphological cytoarchitecture remains unaltered and the defective GABAn remain in the mantle zone until death (*Mash1* ^*−/−*^ mice display a lethal phenotype at birth and *Mgn* ^*−/−*^ mutants die between the second and fourth week). Thus, these similarities suggest the existence of other factors that take over the function of MGN and MASH1 in the vlMB or the existence of different molecular mechanisms.

Protein–protein interactions are intrinsic in eliciting the function of virtually all bHLH factors. To clarify the mechanisms controlling MB-GABAergic neurogenesis and the factors regulating their activity, we performed a yeast 2-hybrid (Y2H) screen with MGN. This assay reported that MASH1 is a direct interactor of MGN. This finding has led us to the discovery of a novel dorsal/ventral molecular mechanism inducing MB GABAergic identity. Our results suggest a new concept regarding two critical families of neurogenic bHLH factors and imply that the MGN and MASH1 factors cooperate synergistically during MB-GABAergic neurogenesis. The combination of gain- and loss-of-function experiments in the developing midbrain showed GABAergic identity in the MB takes place through different dorsal/ventral mechanistic actions at different times.

## Material and Methods

### Y2H screening and 2-protein interaction assays

The Y2H screen, yeast strains, vectors, growth media, and standard methods for manipulating yeast cells were performed according to the manufacturer’s recommendations (Matchmaker GAL4, Clontech) with the following steps: for large-scale screening, the *Mgn* coding sequence (GenBank accession no. DQ294234) was subcloned into a pGBKT7 plasmid (tryptophan, Trp^+^) to express to a transcription factor-binding domain (BD) of the GAL4-DNA vector (bait placed at the C-terminus). A ds-cDNA library was directionally subcloned into the C-terminus of the pGADT7 activator domain (AD) vector (leucine, Leu^+^) using mRNA from E9–E10.5 mouse embryos (Matchmaker library construction kit, Clontech). The 2 *Saccharomyces cerevisiae* yeast strains used in the screen, AH109 and Y187, were auxotrophic for several amino acids (*Trp*; *Leu*; histidine, *His*; and adenosine, *Ade* ^*-*^). The AH109 strain transformed with BD-MGN fusion protein did not grow on SD/*His3* ^*−*^ plates and was negative for β-gal activity, excluding the possibility of host autoactivation.

BD-MGN and all bait plasmids used for the Y2H and 2-protein interaction screen appeared not to be toxic to the yeast cells when plated on SD/*Trp* ^*−*^ plates. Therefore, BD-MGN plasmids were used to screen our primary cDNA library (not amplified). Basal *His3* gene expression was inhibited with 3-amino-triazole (3-AT) in a pilot transformation before performing the large-scale screening experiment. Competent AH109 cells (yeast transformation kit, YEASTMAKER #K1606-1) were co-transformed with bait and prey vectors using the lithium acetate-mediated method. After electroporation, only transformants where protein-protein interaction between the bait and prey took place, activation of His and Ade promoters occurred and were able to grown on synthetic dropout medium (SD) lacking tryptophan (*Trp* ^*−*^), leucine (*Leu* ^*−*^), histidine (*His* ^*−*^) and adenosine (*Ade* ^*−*^) (SD/*Trp* ^*−*^ *Leu* ^*−*^ *His* ^*−*^ *Ade* ^*−*^). Approximately 2.8 × 10^6^ co-transformants were screened and subsequently selected for growth on SD/*Ade* ^*−*^ *His* ^*−*^ *Leu* ^*−*^ *Trp* ^*−*^ medium containing 3 mM of 3-AT. Co-electroporation of the bait and prey vectors resulted in the isolation of 160 putative clones.

In addition to a nutritional selection of the *His3* gene, we performed color-based assay to detect the transcription *LacZ* reporter, because β-galactosidase activity allows a more stringent assay than the *His3* gene in the GAL4 system (less likely to give false positives). After 7–15 days of growth at 30°C on SD/*Ade* ^*−*^ *His* ^*−*^ *Leu* ^*−*^ *Trp* ^*−*^ plates with 3 mM 3-AT (high stringency selection), colonies were replicated onto plates containing X-gal substrate by the colony-lift filter assay. Positive interactors that showed β-galactosidase activity when co-expressed with the bait, but not when co-expressed with the bait-plasmid control, were considered bait-dependent transformants. Co-transformants that survived the HIS3^−^ growth selection and blue-colored colonies were selected.

The identity of the interactors was determined by sequencing. To test paired genes in the 2-protein interaction assay in yeast, the pGBKT7-BD expressing *Mgn*–*Mash1* or *Ngn2* as the bait and the pGADT7 vector harboring the coding sequence of *Mash1*, *Ngn1*, *Ngn2*, *Ngn3*, *and Mgn* as preys were used to transform haploid yeast AH109 (Mat-a) and Y187 (Mat-α) strains, respectively. Yeast strains were mated and diploid cells were plated on SD/*Ade* ^*−*^ *His* ^*−*^ *Leu* ^*−*^ *Trp* ^*−*^ media. To quantify the relative strength of the protein–protein interaction in the 2-protein interaction assay, quantitative β-galactosidase assays were performed on liquid cultures using X-Gal (Sigma #B4252500MG). After cell lysis, a pellet was resuspended in water and mixed with PBS (pH 7.4) containing 500 μg/mL of X-gal, 0.5% agarose, and 0.05% β-mercaptoethanol, followed by incubation at room temperature. The p53/SV40 were diluted 1/5 to adjust the signal to the linear range due to their strong β-galactosidase activity.

Five independent transformants were assayed per interaction pair. The results were normalized against cell density and dilution. In parallel experiments, measurement of lacZ activity by OD was performed using ONPG as a substrate. The reaction was stopped with chilled 1 M Na_2_CO_3_ and absorbance was determined at 420 nm according to the manufacturer’s recommendations (Yeast Protocols Handbook, PT3024-1, Clontech).

### Mouse lines and breeding

*Mgn* and *Mash1* mouse strains and their genotyping have been described previously ([9,13], respectively). The strains were maintained at the Helmholtz Zentrum München. *Mgn/Mash1* compound mutants were obtained from intercrosses of *Mgn* ^*−/+*^ and *Mash1* ^*−/+*^ inbreed mice that had been consecutively backcrossed for eight generations to C57BL/6. Upon crossing double-heterozygous mice (*Mgn* ^*−/+*^ */Mash1* ^*−/+*^ x *Mgn* ^*−/+*^ */Mash1* ^*−/+*^), a myriad of compound mutants could be found. The term “single mutant” is reserved for *Mgn* ^*−/−*^ */ Mash1* ^*+/+*^or *Mgn* ^*+/+*^ */Mash1* ^*−/−*^ genotypes. The term “double mutant” is reserved for *Mgn* ^*−/−*^ */Mash1* ^*−/−*^ genotype. The term “compound mutant” refers to any other genotype in general, otherwise specified. The compound mouse *Mgn* ^*−/+*^ */Mash1* ^*−/+*^ deserves, in this article, a specific mention, given its particular phenotypic traits that differentiate them from the rest of compound mice. Therefore, it is referred as “double heterozygous mice. This study was carried out in strict accordance with the recommendations in the Guide for the Care and Use of Laboratory Animals of the European Union and of the Federal Republic of Germany (TierSchG). The protocol was approved by the Helmholtz Zentrum München Institutional Animal Care and Use Committee (ATV). Surgery was performed under sodium pentobarbital anesthesia, and all efforts were made to minimize suffering.

### Histological analysis

Noon on the day when a vaginal plug was detected was considered E0.5. Embryos were staged precisely by counting somites. Embryos and dissected brains from different stages (E9.5–E18.5) were fixed in 4% paraformaldehyde overnight at 4°C, paraffin embedded, cut on a microtome at 4–6 μm, and hybridized with radioactive ((α-^35^S)-UTP) *in situ* probes (see also oligonucleotides and ISH probes in S1 Material and Methods). Following *in situ* hybridization (ISH), paraffin sections were counterstained with cresyl violet. All analyses were confirmed using 3 embryos from different litters.

### Immunohistochemistry and colocalization

A monoclonal mouse anti-MGN antibody (clone JG35) was raised against the MGN protein (GenBank accession no. ABB96784.1) and purified by protein A/G affinity chromatography. The primary antibodies were as follows: rabbit anti-β-galactosidase (AbD Serotec #AHP1292; 1:100); mouse anti-BRN3a (Santa Cruz #sc-8429; 1:200); mouse anti-calbindin (Swant #300; 1:500); rabbit anti-calretinin (Swant #7699/4; 1:2000); rabbit anti-cleaved caspase-3 (cCASP3; Cell Signalling #9661; 1:1200); rabbit anti-GABA (Sigma #A2052; 1:300); mouse anti-NeuN (Chemicon #MAB377; 1:1000); mouse anti-NKX2-2 (DSHB #74.5A5-c; 1:250); rabbit anti-phospho-histone H3 (pHH3; Upstate #06–570; 1:500); and rabbit anti-tyrosine hydroxylase (TH; Merck Millipore #AB152; 1:150). For colocalization studies in the MB, mouse anti-MASH1 isotype IgG1 (BD Pharmingen #556604; 1:300) and mouse anti-MGN (# JG35; 1:5000) were used. Secondary antibodies were goat anti-mouse IgG-1 Alexa Fluor 594 (Invitrogen #A21125; 1:500) and goat anti-mouse IgG-2b Alexa Fluor 488 (Invitrogen #A21141; 1:500), counterstained with 4′,6-diamidino-2-phenylindole (DAPI) or coupled with horseradish peroxidase (HRP) and detected using the Vectastain ABC Elite Kit (Vector Laboratories/ USA). IHC and ISH paraffin sections were visualized with a Zeiss Axiovert-200M microscope, Stemi SV6 stereomicroscope, and Olympus IX81-Fluoview FV100 confocal laser-scanning microscope.

### Mammalian cell culture and co-immunoprecipitation (Co-IP)

For the 2-protein Co-IP validation assays in mammalian cell culture, HEK293 cells were transiently co-transfected (FuGENE-6 transfection kit; ROCHE #1815091) with the coding region of *Mgn* or *Mash1* (N-terminal FLAG-tagged) and *Mash1* or *Mgn* (N-terminal myc-tagged) subcloned into pcDNA3.1 vectors. The transfected cells were incubated at 37°C for 24–48 h. The pull-down assay was performed using the Protein G immunoprecipitation kit (Sigma **#**IP50). Total protein concentration was measured with the Pierce BCA protein assay kit (Thermo #23227) and the products were analyzed on 10% NuPAGE (Novex #NP0301). The blots were blocked with 4% skimmed milk in TBST, followed by immunoblot analysis with anti-c-myc antibody (Dianova #9E10.3; 1:8000), anti-FLAG antibody (Sigma #F1804; 1:50000), and HRP-conjugated goat anti-mouse IgG (H+L) (Jackson #115-035-003; 1:1000) secondary antibody. For the MGN/MASH1 heterodimer and homodimer Co-IP assays, dMB, vMB, and the rest of the body from E12.5 wild-type (WT) embryos as well MB tissue from E12.5 *Mgn* ^*−/−*^ and *Mash1* ^*−/−*^ mutant embryos were dissected and collected. Brain tissues homogenized in RIPA buffer were lysed in the presence of protease inhibitor cocktail (Complete, Roche #04693124001) with DNases and RNases and then incubated with MASH1 or MGN antibodies covalently coupled and cross-linked with G-beads (Pierce Crosslink Magnetic IP/CO-IP Kit, Thermo scientific #88805). The protein lysate was incubated over night with cross-linked magnetic beads washed with low ionic strength buffer (120 mM NaCl) and eluted with mildly acidic buffer (100 mM glycine-HCL (pH 2.5) for 10 min). The products were verified by classical Western blot analysis using anti-MGN (Abcam; #ab101842; 1:300, anti-MASH1 (BD Pharmingen #556604; 1:300), and peroxidase-conjugated antibodies. Detection was performed using an ECL substrate (Amersham #RPN2232) and exposure to Hyperfilm ECL (GE Healthcare). Mouse embryonic stem (ES) cells were electroporated with a pCDNA3-1 vector harboring the cDNA-coding region of *Mgn* and/or *Mash1* genes. The protein lysate was extracted 24–48 h later for Co-IP assays. Mouse anti-β-actin (Abcam #ab6276; 1:5000) was used as a quality control prior to Co-IP.

### Real-time quantitative PCR (qPCR) and protein quantification

RNA was extracted from 3 E9.5–E10 mouse embryos for each genotype (RNeasy Kit, Qiagen #74104). Residual DNA was further removed from all extracts by DNase digestion and ds-cDNA was synthetized with d(T)_12–18_ oligos (SuperScript, Invitrogen #11904–018). Amplification and real-time measurement was performed on a LightCycler real-time PCR system (Roche) using 10 μL of 2× Fast SYBR Green Real-Time PCR Master Mix (Life Technologies #4385610). The relative expression ratio of *Mgn* and *Mash1* genes among genotypes was computed and analyzed according to the crossing point and threshold values with kinetic PCR efficiency correction method [14]. The *ß-actin* gene was probed to be unaffected among genotypes by comparing the values with other housekeeping controls (*Gapdh*, β-tubuline, and *Pgk1*) and therefore used as a loading control for normalization. The PCR length was 70–100 bp according to the Bustin’s guidelines [15] (MIQE compliant).

### Proliferation and cell death studies

Pregnant females carrying E9.5–E13.5 embryos were intraperitoneally injected with a single BrdU injection (50 μg/g body weight) at 15 min and at 1 h before they were sacrificed. Incorporation and detection of BrdU in cellular DNA were performed with the BrdU labeling and detection kit II (Roche, #1299964). For the terminal deoxynucleotidyl nick end labelling (TUNEL) assay, apoptosis was detected using the *In Situ* Cell Death Detection kit-Fluorescein (Roche, #1684795). Apoptosis in the mouse embryo limbs served as the positive control for the cCASP3 and TUNEL assays. The cells were counterstained with DAPI and analyzed under a fluorescence microscope (Zeiss Axiovert-200M).

### Organotypic explant culture and micro-electroporation

Organotypic WT and *Mash1* ^*−/−*^ mouse anterior neural tube explants (ONTCs) were collected and opened along the dorsal midline prior to electroporation, as described previously [16]. Electroporation assays were performed using a square pulse generator device. Plasmid (*Mgn-IRES-EGFP*) (1 μg/μl in Fast Green, Sigma) was injected into the neuroepithelium using a pressure injector and constantly visualized by fast green. Electroporation was performed by applying 5 pulses of 10 ms at a current of 350 μA with intervals of 50 ms. After electroporation, each explant was cultured *ex vivo* at 37°C up to 24 h and then fixed in 4% paraformaldehyde before further processing for ISH in whole-mount embryos (WISH).

### Cell counting and statistical analysis

For the calculation of NeuN and Calbindin positive cells, we sampled three 0.02 mm^2^ bins in the dorsal, mediolateral, and ventrolateral regions of the MB. A total of three cases were counted in sections for each region. For the calculation of proliferative cells at E10.5 and E12.5, all PHH3+ cells were counted from six slices taken from rostral to caudal MB/group (n = 3). In all cases, the experimenter was blind during sampling, image analysis, data collection and statistical analysis. Statistical analysis was performed with the GraphPad Prism v6.04 software by pair-wise comparisons using Student’s *t*-test and the significance level was taken as *p* < 0.05. Data are represented as mean ± SEM, unless otherwise specified.

## Results

### Identification of MASH1 as the binding partner of MGN protein by the Y2H screen

**Fig 1:**
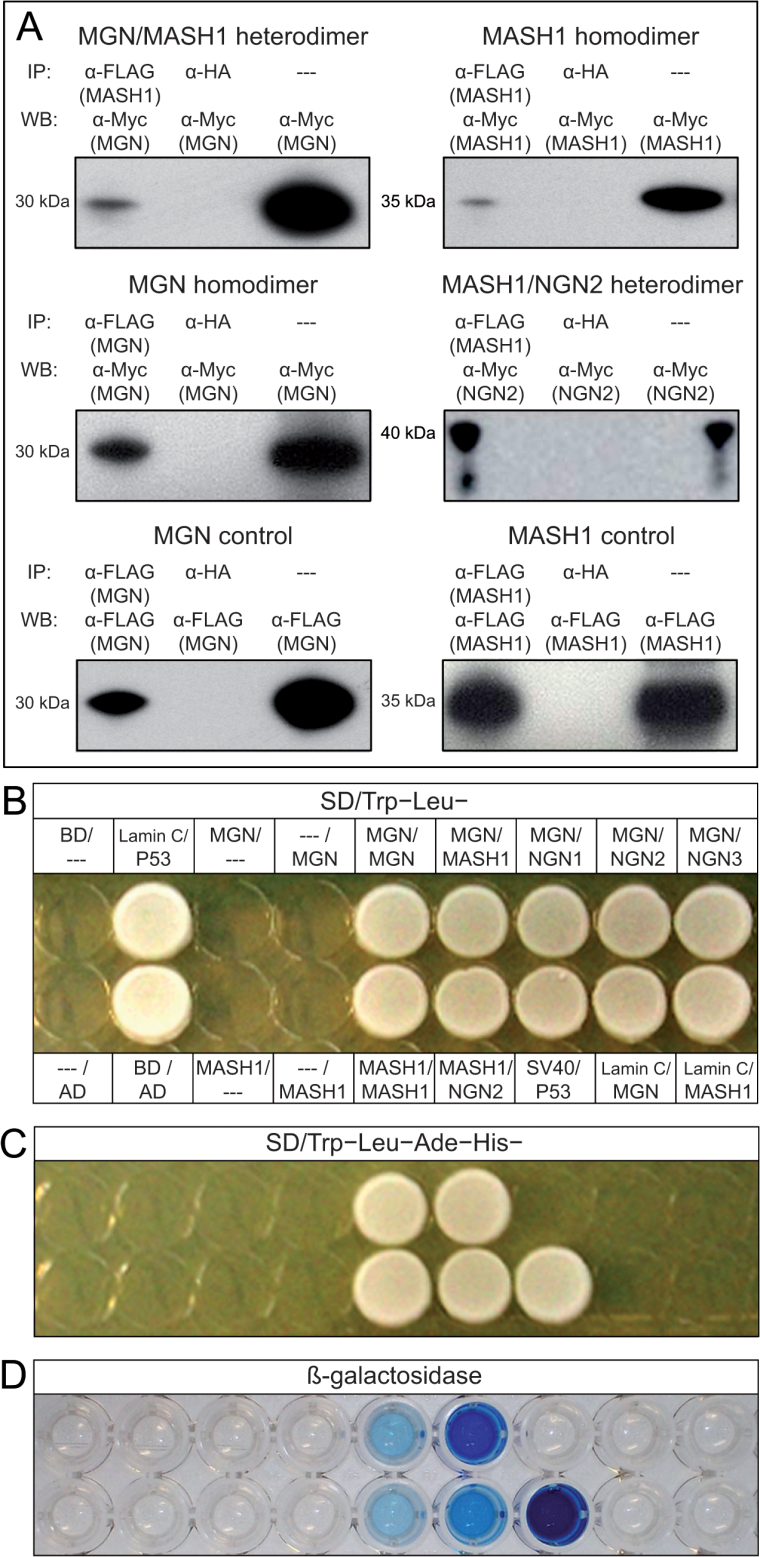
MGN formed homodimers and heterodimers with MASH1 in yeast. **(A)** HEK293 cells were co-transfected, immunoprecipitated (IP), and analyzed by western blotting (WB) with plasmids and antibodies indicated in the IP and WB lines, respectively. The amounts of MGN, MASH1, and NGN2 were verified in total cell lysates (—-) as a control test. Anti-HA antibody (α-HA) was used as a negative control. **(B)** Y2H analysis of the interactions between MGN and different proneural proteins. AH109 yeasts were co-transformed with a bait and prey plasmid (bait/prey) before being plated on synthetic dropout medium (SD/Leu^−^Trp^−^). (**C**) The same yeast colonies were plated in the same order on SD/Ade^−^His^−^Leu^−^Trp^−^ (to select positive protein–protein interactions) and used for the ß-galactosidase activity assay (**D**). Binding domain (BD) alone and activator domain (AD) alone plasmids; BD–human Lamin C provided a control for the AD–prey interaction. P53/SV40 interaction was used as positive control (P53/SV40 was diluted to 1 in 5 in **D**).

To find novel factors involved in the MB-GABAergic pathway, we used MGN as a bait in a Y2H assay to screen a cDNA library of E10.5 mouse embryos to identify protein–protein interactions at the time when MB-GABAergic neurogenesis begins. Expression of the fusion protein in yeast was confirmed (S1 Fig) and the absence of self-activation was verified. Each interaction was further assessed by positive β-galactosidase activity. Protein–protein interactions were confirmed individually with a second Y2H assay by α/a mating between MGN-bait and each segregated positive clone. The 160 clones were sequenced and represented 25 unique proteins (S1 Table). Among the protein interactors obtained in the Y2H assay, MGN and MASH1 factors were identified. As further indication of the validity of the assay, we confirmed binding specificity between MGN and MASH1 proteins using a mammalian cell line. Co-IP assays using MGN and MASH1 epitope-tagged fusion proteins confirmed the data from our yeast screening results (Fig 1A). MGN can form both homodimers (MGN/MGN), as previously reported by *in vitro* experiments in 293E cells [7], and heterodimers with MASH1 (MGN/MASH1). Based on the protein–protein interaction between MGN and the proneural MASH1, we tested if MGN interacted with other bHLH members of the proneural family, such as the neurogenin proteins. The results from the 2-protein assay in yeast and from further tests by Co-IP in mammalian cell cultures revealed that MGN was unlikely to form dimers with the neurogenin proteins NGN1 (*Neurog1*_MGI), NGN2 (*Neurog2*_MGI), and NGN3 (*Neurog3*_MGI) (Fig 1B and 1C), as suggested by our Y2H screen data.

### MGN and MASH1 form heterodimers *in vivo*

*Mash1* was expressed in a manner similar to *Mgn* (i.e., at high levels in the vlMB area at E10.5 when compared with the low levels in the dMB at E12.5). Furthermore, both *Mash1* and *Mgn* have been proved to function as key factors for the specification of dMB-GABAn by loss-of-function experiments in mice [9,10,11]. Therefore, we focused on the role of these two factors and the mechanisms underlying their role in MB-GABAergic neurogenesis.

**Fig 2:**
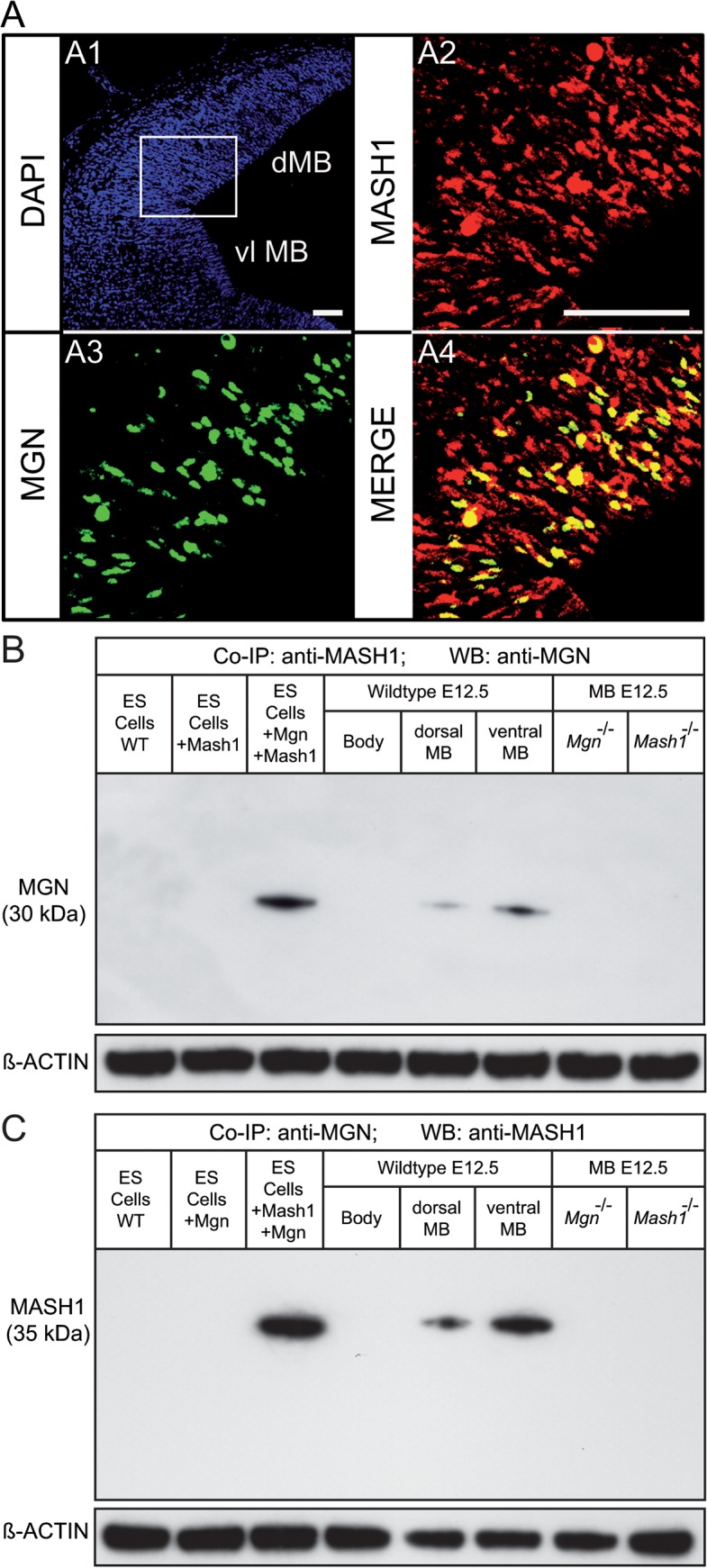
Colocalization and co-immunoprecipitation of MGN and MASH1 in mice. Immunohistochemical staining of coronal sections from E12.5 wild-type mouse embryos. **(A)** Representative confocal photomicrographs of immunohistochemical staining with anti-MASH1 (A2) and anti-MGN (A3) from the dMB (squared area). Nuclei stained with DAPI are shown in blue (A1). A merged picture of A2 and A3 indicating the colocalization of MASH1 and MGN is shown in A4. The MGN^+^ subpopulation represented approximately 50% of the total MASH1^+^ cells of the VZ at this stage. **(B-C)** MASH1 is an interaction partner of MGN in physiological conditions. Endogenous MGN was IP from the dMB and vMB of E12.5 wild-type mouse embryos with anti-MASH1 antibody and detected by western blotting with anti-MGN antibody (**B**) and vice versa (**C**). Lysates from 129SV embryonic stem cells (ES) electroporated with the indicated plasmids, the embryo body (where *Mgn* is not expressed) and the MB from *Mgn* ^*−/−*^ and *Mash1* ^*−/−*^ mutants were used as controls. Equal input and quality of the protein lysate prior to immunoprecipitation was shown by immunostaining with anti-ß-ACTIN antibody. Scale bars: 100 μm.

Colocalization studies between MGN and MASH1 were mandatory because of the so-called time/space constraints, according to which two proteins may never be in close proximity to each other within the cell even though they are otherwise able to interact. Given that MGN and MASH1 colocalize in most vlMB cells [8] and that the GABAergic phenotype of single knockout mice is circumscribed in the dMB, we studied colocalization in that area. Although the MASH1 domain was broader (also found in postmitotic neuronal progenitors) than the MGN domain (restricted exclusively to mitotic neuronal progenitors of the VZ), all MGN^+^ cells were positive for MASH1 in VZ of the dMB at E12.5 (the onset of dGABAn) (Fig 2A). Co-IP analysis of endogenous protein extracts derived from WT using specific antibodies recognizing each protein indicated that MGN and MASH1 form heterodimers in the dorsal and ventral aspect of the MB at the onset of GABAergic neurogenesis *in vivo* (Fig 2B and 2C). This interaction does not take place in single mutants. Embryonic stem (ES) cells were used as control to validate the protein-protein interaction observed in the immunoprecipitation analysis from mouse brain tissue. Heterodimer formation was visualized only in lysed ES cells expressing MGN and MASH1 proteins. Contrary, MGN/MASH1 heterodimerization does not take place in wild-type cells or ES cells expressing either *Mgn* or *Mash1*, indicating a specific interaction.

### MGN and MASH1 heterodimers are needed for proper dMB-GABAn specification *in vivo*

**Fig 3:**
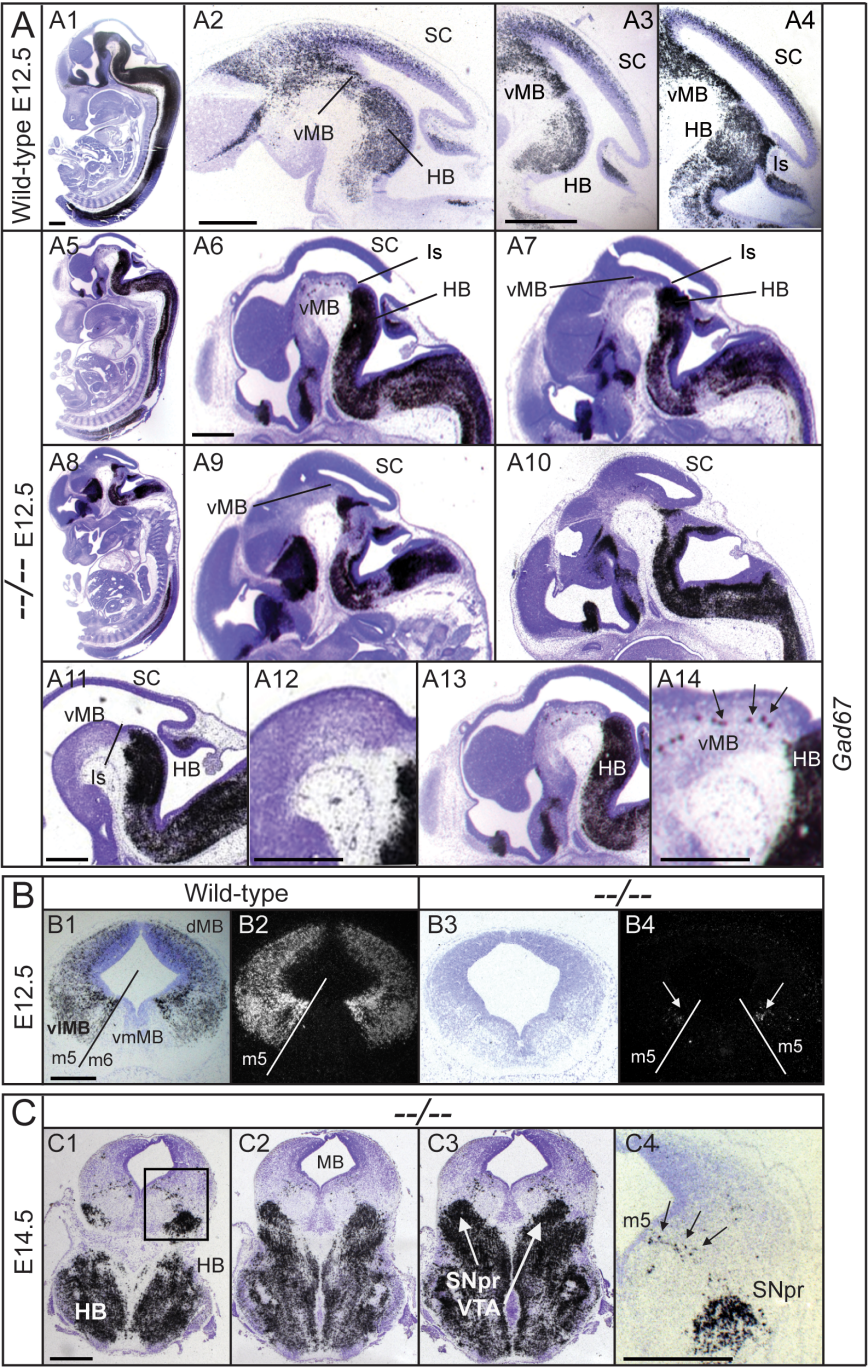
*Mgn* and *Mash1* were necessary and sufficient to induce GABAn in the MB. Phenotypic analysis of *Mgn* ^*−/−*^ *Mash1* ^*−/−*^ (*−−/−−*) mice by ISH with the *Gad67* riboprobe on sagittal **(A)** and coronal **(B-C)** sections, showing the mesencephalon at E12.5 and E14.5 from both wild-types (A1–A4; B1 and B2) and double mutants (with B5–B7 being representative slides from anterior to posterior). The arrows in A14, B4, and C4 indicate the presence of few *Gad67* cells in the m5 domain (m5) at E12.5 and E14.5. Scale bars: 500 μm. Abbreviations: HB, hindbrain; Is; isthmus; SC, superior colliculus; SNpr, substantia nigra pars reticulata; vMB, ventral midbrain; VTA, ventral tegmental area.

**Fig 4:**
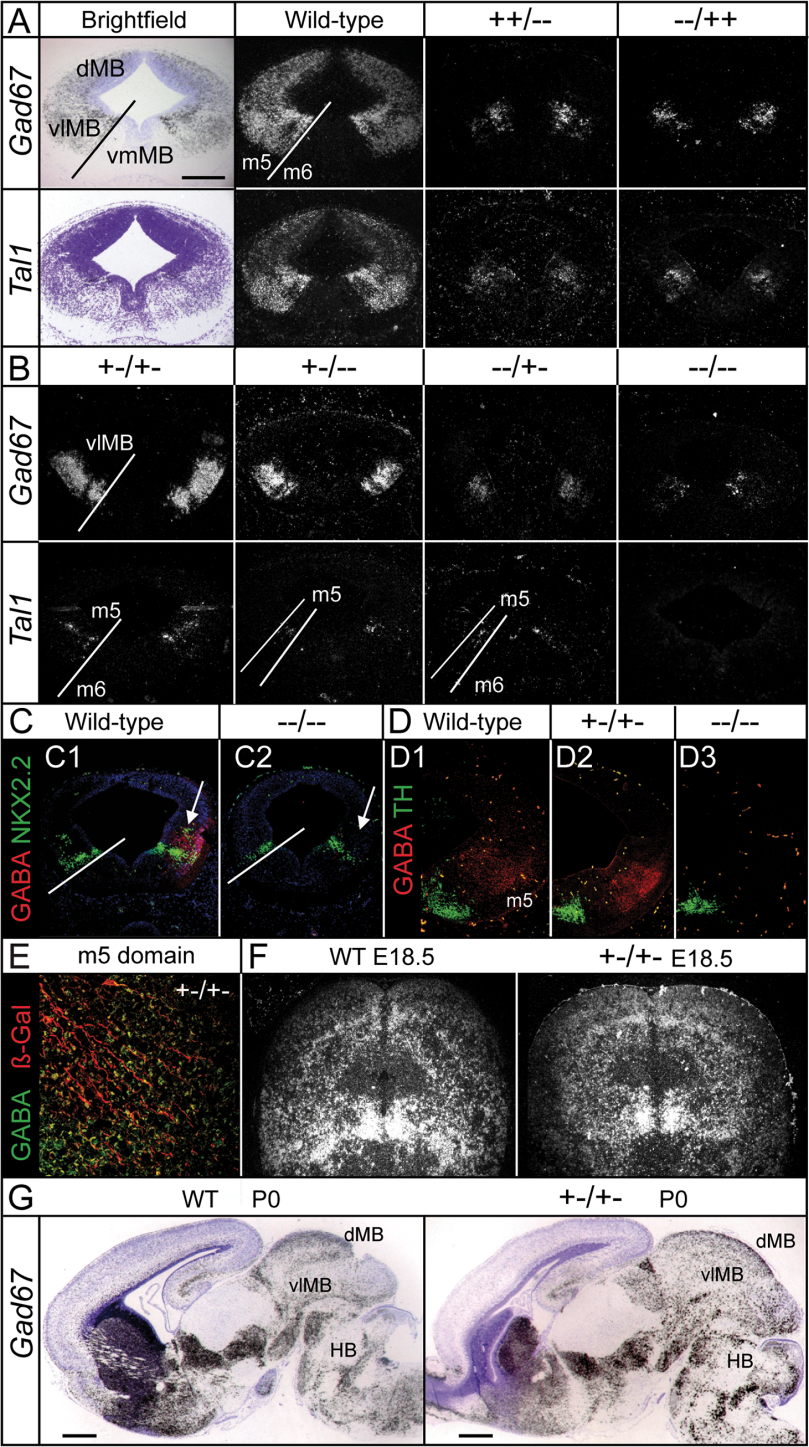
MB GABAergic pathway was severely compromised in the double and compound mutants. Expression of GABAergic-specific genes on the coronal sections of E12.5 in wild-type and single knockout embryos (**A**), as well as in compound and double knockout mutants (**B**). MB GABAergic induction occurred in double heterozygous mice based on the presence of GABAergic markers (*Gad67* and *Tal1*). (**A-E**) The GABA phenotype observed at E12.5 in the vlMB of *Mgn* ^*−/−*^ *Mash1* ^*−/−*^ (*−−/−−*) mice (**C2** and **D3**) was recovered in *Mgn* ^*+/−*^ *Mash1* ^*+/−*^ mice (**D2** and **E)**. The intermediate zone of m3 domain is magnified in **E**. GABAn of double heterozygous embryos were maintained at E18.5 **(F)** and until postnatal death P0 **(G).** DAPI, NKX2-2, TH, and β-Gal staining were used as morphological markers). Arrows indicate the loss of postmitotic NKX2-2 neuronal subpopulation in the m2 domain, but not in the m4 domain (**C**). Scale bars: 500 μm in A and G, and 50 μm in E.

**Fig 5:**
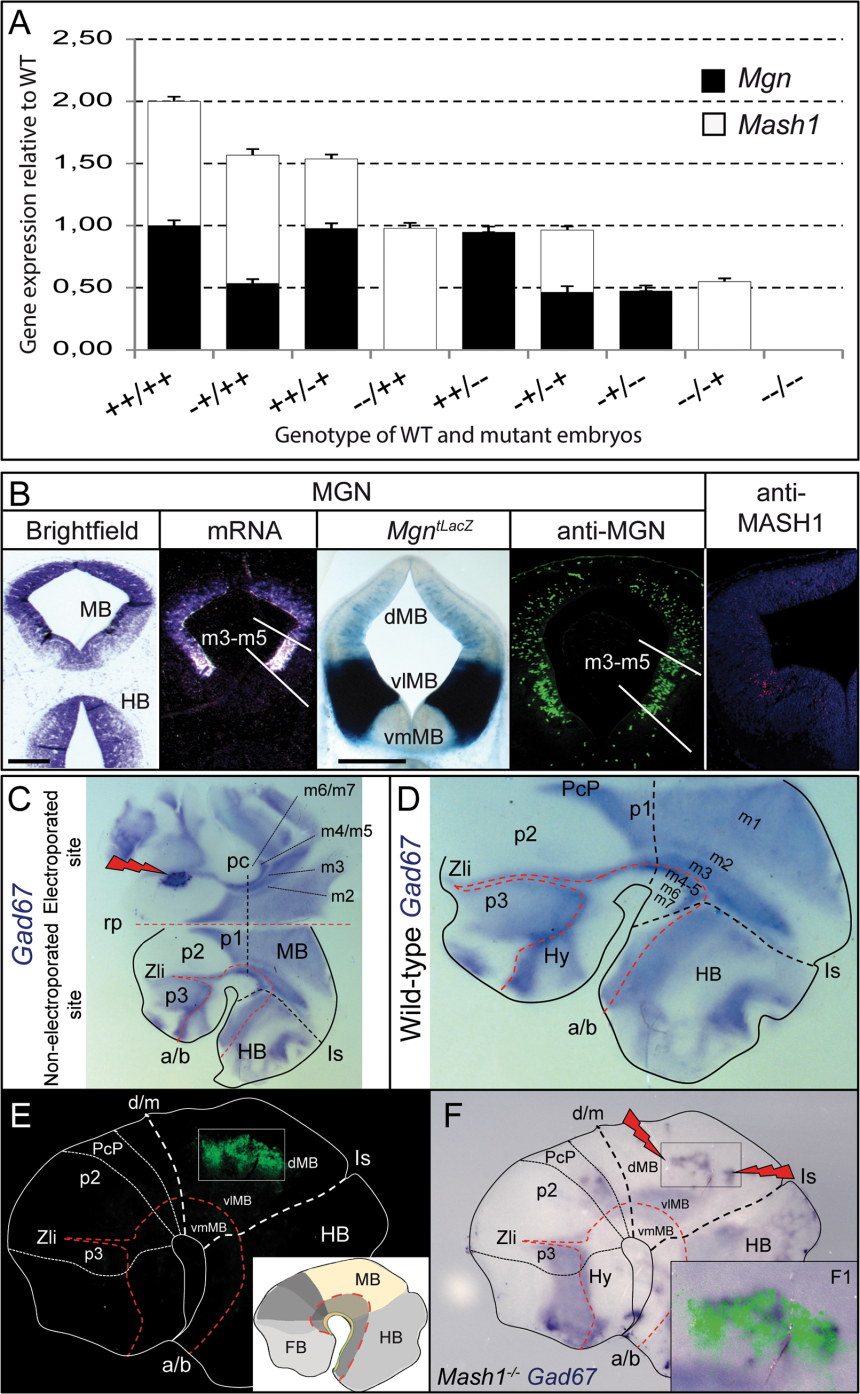
Endogenous and ectopic expression of *Mgn*. **(A)** Relative expression levels of *Mgn* and *Mash1* in mutant to wild-type mice. The bar height and error bars show the mean and standard deviation, respectively. **(B)** *Mgn* mRNA was expressed at high levels in the vlMB of E12.5 mouse embryos compared with the low level of expression in the dMB determined by ISH, LacZ staining of *Mgn* ^*tLacZ*^ mice, and IHC of wild-type embryos with the anti-MGN antibody on coronal sections. The lines indicated the borders of m3-m5 domains. MASH1 protein is also expressed at higher levels in the vlMB compared with its dorsal expression, as determined by IHC with anti-MASH1 antibody. **(C)** WISH was performed with *Gad67* probe in ONTC of wild-type E11.5 embryos with *Mgn* cDNA electroporated in one of the sides (right side), whereas the other side served as a control (left side). The neural tube is opened along the ventral anterior-posterior axis. Lightning bolt icons point to the ectopic expression of *Gad67* after *Mgn* electroporation. Subdomains are shown according to the prosomeric model [21]. (**D**) WISH was performed with a *Gad67* probe in the ONTCs of E11.5 wild-type mouse embryos after 10 h of incubation. The red dashed line delineates the alar/basal (a/b) boundary. The black dashed lines delineate the transversal neural tube domain boundaries. (**E and F**) E11.5 *Mash1* ^−/−^ ONTCs 24 h after micro-electroporation in the mesencephalic alar plate with the Mgn-IRES-EGFP plasmid. **(E)** A fluorescence signal showing the electroporated location. **(F)** Induction of *Gad67* expression after electroporation of *Mgn* cDNA in *Mash1* ^−/−^ background embryos. (**F1**) A merged profile of the fluorescence signal and *Gad67* induction. Scale bars: 500 μm. Abbreviations: a/b, alar/basal plate boundary; d/m, diencephalic–mesencephalic boundary; Hy, hypothalamus; Is, isthmus; p1–p3, prosomeres 1 to 3 in diencephalon; PcP, precommissural domain in the pretectum (p1); rp, roof plate; Zli, zona limitans intrathalamica.

The analysis of *Mgn* single knockout mice (*Mgn* ^*−/−*^) revealed a GABAergic phenotype very similar to that of the *Mash1* single knockout mice (*Mash1* ^*−/−*^). Both single mutants have complete depletion of GABAn in the SC and induction of dMB-GABAn does not take place at any time during development [9,10,11]. However, induction of vlGABAn does occur and, although a slight delay in neurogenesis is observed between E10.5 and E14.5, vlMB-GABAergic induction is clearly observed. A puzzling phenotypic feature of *Mgn* and *Mash1* single mutants is that the GABAergic phenotype is circumscribed to the domain where *Mgn* and *Mash1* are expressed at low levels compared with their ventrolateral expression. Therefore, a key developmental question arose: is there a third redundant factor specifying vlMB-GABAn at E10.5 or does MB-GABAergic neurogenesis rely on different mechanisms? Concomitant loss of function of *Mgn* and *Mash1* (*Mgn* ^*−/−*^ *Mash1* ^*−/−*^) revealed that in contrast to *Mgn* and *Mash1* single mutants, GABAergic fate is severely compromised in *Mgn* ^*−/−*^ *Mash* ^*−/−*^ embryos and most GABAn of the MB are absent (Fig 3A). Time-course analysis showed that the induction of GABAn does not take place at any time during MB development, as determined by the absence of the GABAergic markers *Gad65* (*Gad2*_MGI), *Gad67* (*Gad1*_MGI), and GABA in *Mgn* ^*−/−*^ *Mash1* ^*−/−*^ embryos. Therefore, complete ablation of GABAn was observed in both the dMB and vlMB, and only few GABAn were detected in the vlMB m5 domain of double mutant mice (Fig 3B4 and 3C4). This phenotype occurred with 100% penetrance and persisted to death, indicating that there is no other code involved in their specification except for a small number of cells located within the m5 domain (intermediate basal plate). However, double heterozygotes (*Mgn* ^*+/−*^ *Mash1* ^*+/−*^) showed normal GABAergic induction (dorsally and ventrally) compared with WT animals (Fig 4B, 4D2 and 4E) and it is maintained until postnatal stage 0 (P0) (Fig 4F and 4G). We therefore tested whether or not a compensatory mechanism existed between the levels of mRNA expression of *Mgn* and *Mash1* in double heterozygous mice. Reverse transcription-qPCR analysis revealed that *Mgn* mRNA expression was not upregulated in *Mash1* ^*−/−*^ mice and vice versa (Fig 5A). This indicated that the expressions of *Mgn* and *Mash1* were independent and occurred without compensatory mechanisms in single or compound mutants. Therefore, MGN/MASH1 heterodimers appear necessary for dMB-GABAn induction. This novel finding prompted us to hypothesize that heterodimer formation may have a higher efficiency than homodimer formation in dMB-GABAergic specification. One appealing feature of the Y2H system is that the strength of interaction predicted by the 2-hybrid approach essentially correlates with that determined *in vivo* [17]. We quantified the difference in relative strength between heterodimers and homodimers in a double manner. On one hand, we increased the stringency of the growing conditions by adding 3-AT to the media, an inhibitor of HIS3 synthesis, to discriminate different degrees of affinity interaction. On the other hand, we measured β-galactosidase activity in liquid cultures because of its higher sensibility and reproducibility. The 3-AT analysis showed that only yeasts forming MGN/MASH1 heterodimers were able to grow in higher levels of 3-AT, whereas yeasts forming MGN/MGN or MASH1/MASH1 homodimers were not (Figures A, B and C in S2 Fig). Consistent with these results, the β-galactosidase assays showed that the strength of the MGN/MASH1 protein interaction was 4.4 and 4.0 times higher than that for the MGN/MGN and MASH1/MASH1 homodimers, respectively (Figures D and E in S2 Fig, and Fig 1D). Thus, MGN/MASH1 heterodimers were more efficient in activating *HIS3* and *LacZ* gene transcription than their homodimers.

We next investigated why MGN/MASH1 heterodimers, but not their homodimers, are sufficient for GABAergic induction. Phenotypic analyses of single mutants (where heterodimer formation cannot take place) and double heterozygous mice revealed that the presence of a single WT allele from each locus was sufficient to promote dMB-GABAergic induction, whereas two *Mgn* or two *Mash1* alleles alone were not. These observations suggested that heterodimer formation is essential for proper d-GABAergic neurogenesis but not for vl-GABAn, where only a single allele from the *Mgn* or *Mash1* loci was sufficient to trigger GABAergic induction. Therefore, MGN and MASH1 cooperate synergistically for dMB-GABAergic neurogenesis.

### A dose-dependent mechanism for vl-GABAn

The absence of GABAn in the double knockout mice suggested that dorsal and vlMB-GABAergic induction were controlled by the same codes, raising questions about the intrinsic cell mechanisms that govern the differential development of GABAn in the dMB and vlMB. Dorsally, analysis of compound mice showed that both genes do not have a predominant role over the other and that both alleles contribute equally to the MB-GABAergic phenotype. One allele per locus was required for the formation of MGN/MASH1 heterodimers and for the specification of d-GABAn. Ventrolaterally, the presence of two alleles (regardless of whether they come from the *Mgn* or *Mash1* locus) was sufficient to determine the GABAergic fate; that is, heterodimers were not required because GABAergic induction took place in *Mgn* ^*+/+*^ *Mash1* ^*−/−*^ or *Mgn* ^*−/−*^ *Mash1* ^*+/+*^ mice. Furthermore, the presence of a single allele (*Mgn* ^*+/−*^ *Mash1* ^*−/−*^ or *Mgn* ^*−/−*^ *Mash1* ^*+/−*^ mice) was sufficient to trigger GABAergic induction in the vlMB, although a strong reduction in GABAn was observed (Fig 4B). Thus, the severity of the vlMB-GABAergic phenotype increases in a dose-dependent manner with the number of mutant alleles from the *Mgn* and *Mash1* loci. The phenotypic severity culminates in the absence of GABAn in the double knockout mice (Fig 4C2 and 4D3), except for a small number of residual GABA^+^ cells in the ventrolateral m5 domain. These data support a model that requires both MGN/MASH1 heterodimers and dose dependency as mechanisms operating at the dMB and vlMB, respectively. Consistent with this finding, the high level of *Mgn* expression observed in the vlMB (Fig 5B) may compensate for the lower efficiency of homodimers compared with that of heterodimers. MAHS1 protein is also not evenly distributed throughout the MB but highly expressed in the vlMB domain (Fig 5B). This result is in good agreement with published data from Partanen’s group [11] where *Mash1* mRNA expression is markedly higher in the vlMB compared with its dorsal expression. This hypothesis was later tested using gain-of-function experiments. Ectopically expressed *Mgn* cDNA into organotypic cultures of WT mouse embryos at E10.5 induced *Gad65/67* expression (two specific and independent enzymes for GABA synthesis) throughout the nervous system (Fig 5C; additional data not shown). The ectopic *GAD65/67* expression observed in mouse brain regions upon *Mgn* electroporation suggests that *Mgn*, when expressed at high levels, does not need to cooperate with additional cell type-specific transcription factors to execute its function in a cell-autonomous manner. Moreover, we investigated if high expression of *Mgn* cDNA electroporated into the dMB of *Mash1* ^−/−^ embryos can initiate GABAergic cell fate in the absence of *Mash1* (and therefore in the absence of MNG/MASH1 heterodimers). In the *Mash1* ^−/−^ E12.5 embryos electroporated with *Mgn* cDNA, the GABAergic fate was induced as determined by the *in novo* expression of *Gad65/67* (Fig 5F).

### Impaired neurotransmitter specification in the d/vlMB of *Mgn* ^−/−^ *Mash1* ^−/−^ mice

**Fig 6:**
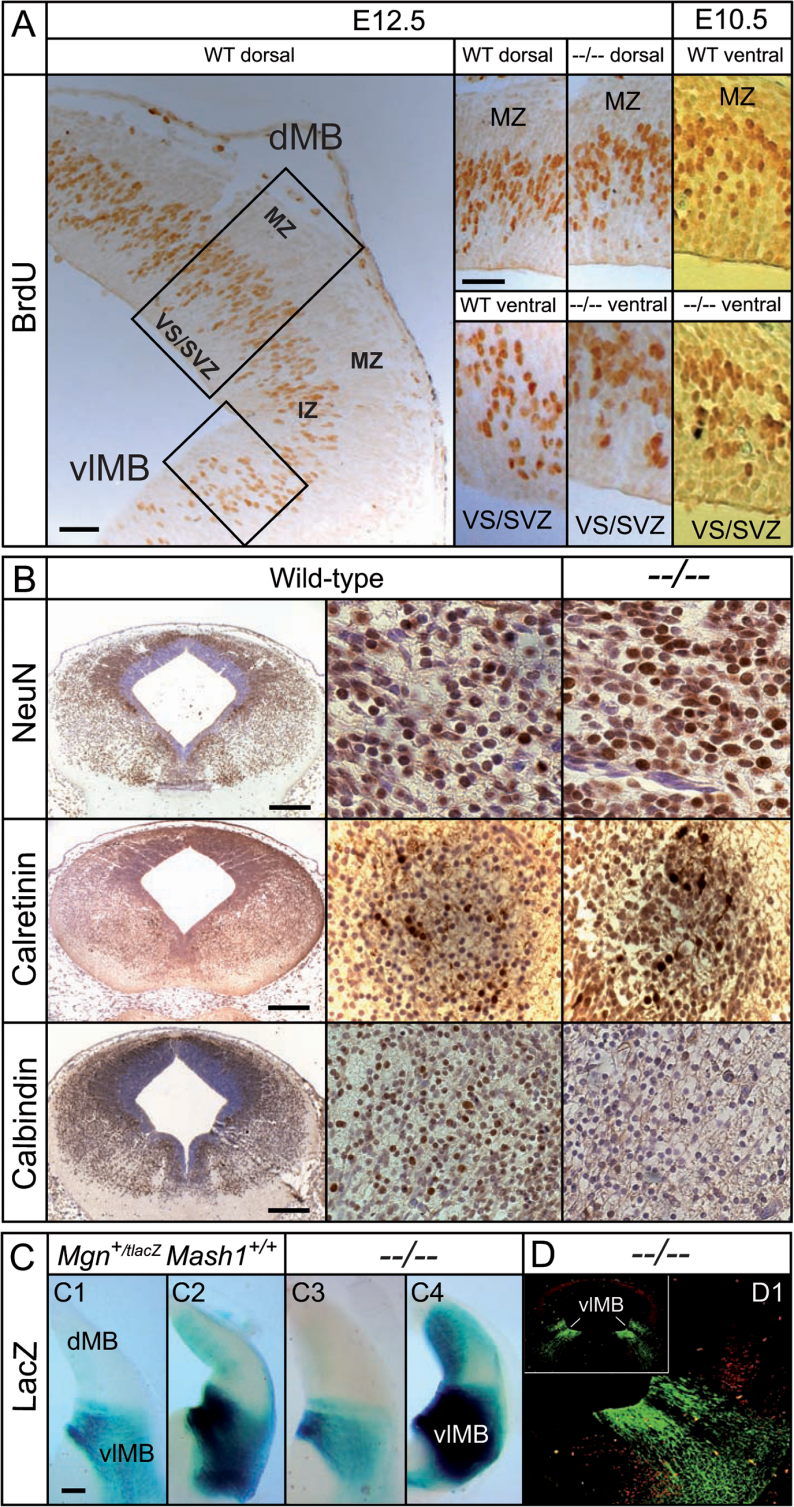
Depletion of dMB and vlMB GABAn in the *Mgn* ^*−/−*^ *Mash* ^*−/−*^ mice occurred through GABAergic identity failure. **(A)** Immunohistochemical staining of BrdU on coronal sections showing the distribution of BrdU^+^ cells in the developing dMB and vMB of wild-type (WT) and double mutant mice (*−−/−−*). **(B)** Neuronal cell density and cytoarchitectural analysis on the coronal sections of E12.5 embryos using NeuN and the Ca^2+^-binding proteins Calretinin and Calbindin, respectively. There were no significant difference between mean WT and double mutant (NeuN: dorsal WT = 64.22±2.08; dorsal *−−/−−* = 61.33±2.69; n = 18; p = 0.40. Medial WT = 52.28±3.60; medial *−−/−−* = 56.72±1.97; n = 18; p = 0.29. Ventrolateral WT = 46.44±2.36; ventrolateral *−−/−−* = 43.67±2.52; n = 18; p = 0.43); (Calbindin: dorsal WT = 43.28±6.20; dorsal *−−/−−* = 37.50±5.54; n = 18; p = 0.49. Medial WT = 52.22±5.32; medial *−−/−−* = 46.56±2.69; n = 18; p = 0.35. Ventrolateral WT = 47.39±3.19; ventrolateral *−−/−−* = 46.17±3.92; n = 18; p = 0.81). **(C)** *Lac*Z staining of coronal sections from double mutants and controls at E12.5; C2 and C4 show prolonged staining times. Owing to the long half-life of lacZ protein, signal can also be seen in the intermediate and mantle zones. **(D)** Immunohistochemical staining with anti-LacZ (in green) and anti-BRN3a (*Pou4f1*_MGI) (in red, as a morphological marker) antibodies that shows radial migration of GABA-defective neurons through the intermediate zone (IZ) toward the mantle zone (MZ) in double mutants. Scale bars: 100 μm in A, and 200 μm in B-C.

The depletion of GABAn engendered the need for cell proliferation, programmed cell death, and cytoarchitectural studies to investigate the cellular basis of the lack of d/vl-GABAn in double knockout mice. No reduction in cell proliferation was identified in the d/vlMB of double mutants at E10.5–E14.5 as revealed by immunohistochemical staining of mitotic cells surrounding the lumen of the neural tube with pHH3 (Figure A in S3 Fig). Cell death was analyzed by IHC to detect cleaved caspase 3 (cCASP3). There was no evidence of increased apoptosis in the MB of *Mgn* ^**−***/***−**^ *Mash* ^**−***/***−**^ embryos when compared with the littermate controls at the time of GABAergic neurogenesis (Figure B in S3 Fig). We next confirmed the results of proliferation and apoptosis using BrdU incorporation and TUNEL assays, respectively. New cells from VZ, time-traced with BrdU, showed no proliferation deficits in the MB of double mutants, and the BrdU-labeled progenitors migrated radially to the mantle zone, as in the littermate controls (Fig 6A). TUNEL assays revealed no apoptotic cells among genotypes on serial samples of coronal sections at E10.5–E13.5 (Figure C in S3 Fig). Furthermore, in the developing MB of double mutants, cytoarchitecture studies revealed no significant difference in the cell density of differentiated neurons within the intermediate and mantle zones (compared with WT mice. This was determined by immunocytochemistry for Neuronal Nuclei (NeuN), a specific marker of postmitotic neurons in vertebrates (Fig 6B). Calcium-binding protein (calretinin and calbindin) marking of subpopulations of GAD^+^ cells in differentiated GABAn indicated that the MB-GABAergic cytoarchitecture of double mutants is not affected at the gross morphological level (Fig 6B). Moreover, we traced lacZ^+^ cells outside the VZ neuroepithelium, expressed under the endogenous *Mgn* regulatory elements in mice with Tau-lacZ knocked in the *Mgn* locus (*Mgn* ^*TlacZ/TlacZ*^ *Mash1* ^*−/−*^ mice). Consistent with previous studies in single knockout mice [9], the same pattern of lacZ staining was observed in compound and double mutants, showing that the *Mgn* ^*−/−*^ *Mash* ^*−/−*^ lacZ-stained cells were positioned in stream-like routes and initiated their radial migration toward the marginal zone through the intermediate zone (Fig 6C and 6D) as neurogenesis ensued [18]. MGN and MASH1 pathway control GABAergic neurotransmitter identity without regulating neurogenesis.

### MGN and/or MASH1 dimers essentially activate the same MB-GABAergic neurogenesis pathway

We looked for downstream GABAergic markers to explore if MGN or MASH1 homodimers in the corresponding single knockout conditions were able to activate equivalent genetic programs compared with the MGN/MASH1 heterodimers present in WT animals. Genetic analyses of specific markers of the GABAergic pathway suggested that the MGN/MASH1 heterodimer and homodimers essentially activate the same pathway. Expression of postmitotic GABAergic terminal selector genes were largely downregulated. Thus, the zinc-finger *Gata2*, *Gata3*, and the bHLH *Tal1* transcription factors, which are essentially co-expressed in the MB GABAn at E10.5-E12.5, follow the dorsal/ventrolateral *Gad65* and *Gad67* phenotypes of single, double, and compound knockout mutants. In particular, the dorsal expression of *Gata2*, *Gata3*, and *Tal1* is donwregulated in single, double, and compound mice, except for the double knockout heterozygotes, where MGN/MASH1 heterodimers are present (Figures A and B in S4 Fig, Fig 4A and 4B, respectively). Ventrally, their downregulation follows the dose-dependent mechanism according to the *Mgn* and *Mash1* genotype and their expression is also absent in the double knockout conditions, showing a total dependence on *Mgn* and *Mash1* genes (Figures A6 and B9 in S4 Fig). Another observation from of our studies is that *Gata2*, *Gata3*, and *Tal1* expression occurred only in few cells in the m5 domain of double knockout mutants in the same subdomain as *Gad65/67* expression. The GABAergic related LIM1 (*Lhx1*_MGI) transcription factor is also misregulated in the vlMB. *Lim1* expression depended on the MGN/MASH1 heterodimers in the m3 and m4 domains only, because LIM^+^ cells were largely absent in these subdomains of all compound mutant mice where heterodimer formation was not possible, and was independent of *Mgn* and *Mash1* expression in the m5 and m6 domains, because normal *Lim1* expression occurred in the double mutants (Figure A in S5 Fig). Unlike *Tal1* expression, which was absent in the double mutants, *Tal2* expression was totally dependent on the MGN/MASH1 heterodimers in the m5 domain, yet independent of *Mgn* and *Mash1* expression in the m1–m4 domains (Figure A in S6 Fig).

Because TAL factors, particularly TAL2, appear to operate as postmitotic selectors between GABA and glutamatergic neurons [19], we investigated transpecification of defective GABAn. Consistent with the GABAergic to glutamatergic neurotransmitter switch observed in single knockouts [10,11], upregulation of *Vglut2* (Slc17a6_MGI)-expressing cells can also be observed in the dMB of *Mgn/Mash1* double mutants, concomitantly with a delay in neurogenesis at E12.5. Ventrolaterally, upregulation of *Vglut2* expression depended on *Mgn*; however, it was not dependent on either *Mash1* expression or in the formation of MGN/MASH1 heterodimers, because *Mash1* single mutants and *+−/−−* compound mice showed no upregulation of *Vglut2*, respectively (Figure B in S6 Fig).

The observation that *Mgn* and *Mash1* both control equivalent programs of MB-GABAergic neurogenesis during this stage of development should not be taken to imply that *Mgn* and *Mash1* have the same function. The ectopic expression of *Vglut2* depending on *Mgn* but not on *Mash1* suggest that these factors have also unique targets. On the other hand, beyond the GABAergic pathway, these particular bHLH codes have distinct roles during development.

## Discussion

Finding the factors dictating neuronal specification and concomitantly understanding the molecular mechanisms and functional interactions between these factors is essential for cell therapy strategies. *Mgn* and *Mash1* are the earliest determinants of GABAergic identity in the d/vlMB at E10.5–E12.5. The 4 alleles have crucial roles in acquiring the GABAergic phenotype, and none has a dominant effect over the other. The complete loss of dMB GABAn in the *Mgn* ^*−/−*^ mice implies that MASH1 homodimers are insufficient to induce the GABAergic fate in the dMB. Likewise, MGN homodimers are also insufficient to induce GABAergic neurogenesis in the dMB of *Mash1* ^*−/−*^ mice. However, double heterozygotes carrying one allele from each locus are able to trigger dMB-GABAergic induction, whereas single knockout mice are not (although still harboring the same number of total alleles).

The 3-AT inhibition experiments and lacZ staining in yeast suggest that a synergistic cooperation probably occurs by increasing the strength of the interaction between the two factors. The higher efficiency of MGN/MASH1 heterodimers compared with homodimers in activating the target genes in the yeast assay may explain the induction of dMB-GABAn despite the low expression levels of *Mgn* and *Mash1*. No downregulation of either *Mgn* or *Mash1* is responsible for the lack of d-GABAn in single knockout mice, yet the synergistic cooperation is also observed in physiological conditions because heterodimers are more efficient at inducing biological responses. This was illustrated by the GABAergic phenotype of compound mutants.

It is noteworthy that both MGN and MASH1 can interact with other bHLH factors and transcription factors. Therefore, we cannot rule out that other protein interactors with either MGN or MASH1 may contribute, to some extent, to the MB-GABAergic induction. However, the observation that the MB of double mutant mice was devoid of GABAn (ventrally and dorsally at E10.5 and E12.5, respectively) indicates that MGN and MASH1 are the critical factors that induce all GABAn originating within the MB, except for a few cells found in the m5 domain. The genetic pathway providing GABAergic neuronal identity to this small subpopulation of GABAn in the m5 domain needs to be elucidated. Nevertheless, we claim that the two major players are MGN and MASH1 proteins, acting dorsally and ventrolaterally by two different mechanisms.

### Different dorsal/ventrolateral mechanisms for GABAergic neurogenesis operating at MB

Two genes, *Mgn* and *Mash1*, function as selectors of GABAergic identity in the vlMB during the first wave of GABAergic neurogenesis at E10.5. This occurs through a mechanism different from that operating in the dMB during the second wave of GABAergic neurogenesis at E12.5. From the comparative phenotypic analysis of *Mgn* and *Mash1* in single, double, and compound mutant mice, we make the following conclusions. First, neither MGN nor MASH1 homodimers can compensate for the loss of MGN/MASH1 heterodimer function in the dMB, possibly because of the low expression levels of *Mgn* and *Mash1* mRNAs in this domain. Second, MGN/MASH1 heterodimers have an instructive role in specifying the identity of dMB-GABAn. It is possible that the higher efficiency of MGN/MASH1 heterodimers over homodimers compensates for the low levels of *Mgn* and *Mash1* expression in the dMB compared with the vlMB. Third, either MGN or MASH1 homodimers are sufficient to trigger vlMB-GABAn at physiological protein levels. Finally, although a gradual phenotype appears in a dose-dependent manner in compound mice, MGN/MASH1 heterodimers are not needed for the induction of vl-GABAn (heterodimer formation is prevented in single knockout mice). In the absence of MGN, MASH1 can take over the function of determining GABAergic cell fates, but only in the vlMB, and vice versa. This is because at these developmental stages (E9.5–10–5), the high level of expression probably circumvents the lack of MNG/MASH1 heterodimer formation in single mutants.

**Fig 7:**
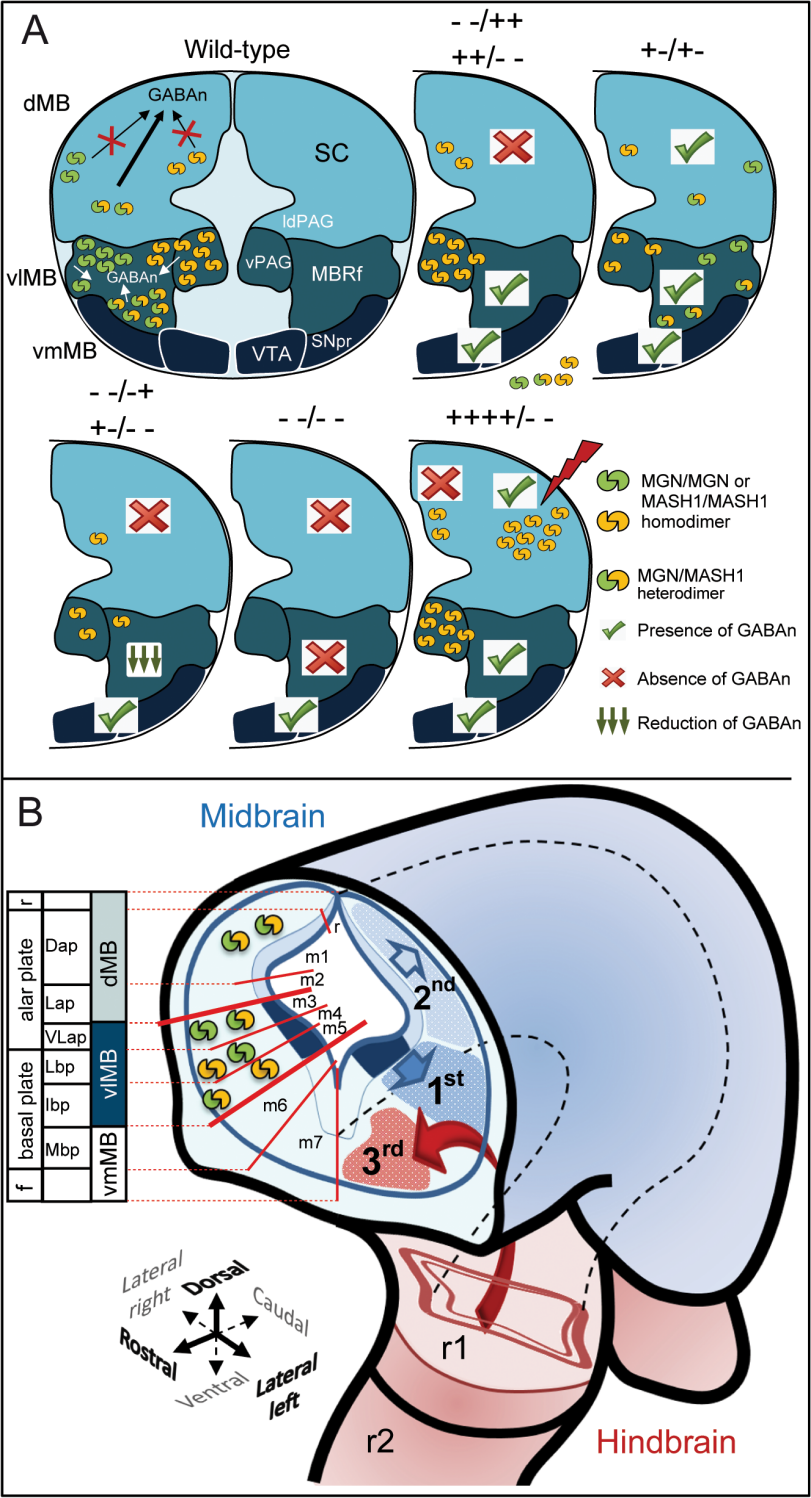
Summary model of the dorsal/ventral mechanisms underlying MB GABAergic neurogenesis. **(A)** Two genes, *Mgn* and *Mash1*, functioned as selectors of GABAergic identity in the MB through different dorsal/ventral mechanistic actions at different times. The MGN/MASH1 heterodimers (green shape coupled with yellow) appear essential for the induction of dorsal GABAn (m1 and m2 domains) that generate GABAn in the colliculi, laterodorsal periaqueductal gray area (ldPAG) and the ventrolateral periaqueductal gray area (vPAG). MGN or MASH1 homodimers (shapes coupled with the same color) are not sufficient to trigger GABAergic identity, and synergistic cooperation between MGN and MASH1 factors to form heterodimers is essential for the acquisition of dorsal GABAergic identity. The mechanism operating in the vlMB area (m3, m4, and m5 domains), which gives rise to the ventral PGA and reticular formation nuclei (MBRf) GABAn, appeared to be different from that in the dorsal area. In this model, the MGN/MASH1 heterodimer is not a prerequisite for the induction of ventrolateral GABAn, with either homodimer being sufficient alone and following a dose-dependent mechanism. The GABAergic phenotype observed in mutant mice is depicted with a green tick or red cross to denote the presence or absence of GABAn, respectively. **(B)** MB GABAn arise during three waves of neurogenesis, at both specific developmental times and specific locations. The earliest wave of GABAergic neurogenesis (1^st^) starts at E10.5 in the presence of *Mgn* and/or *Mash1*, without preference. The second phase (2^nd^) takes place two days later at E12.5 in the dMB. The third population (3^rd^), corresponding to the GABAn of the SNpc and VTA, appears at E14.5. These different mechanisms define a new model for dorsal and ventrolateral GABAergic neurogenesis. Red lines delimitate the embryonic MB into seven subdomains (m1-m7) along the dorsal/ventral axis, characterized by the expression of specific transcription factors [10] and their corresponding MB plates, established by [22]. (*−−/++*; *++/−−*) stand for *Mgn* and *Mash1* single knock-out embryos, respectively. (*−+/−+*; *−−/−+*; *−+/−−*) stand for compound embryos; (*−−/−−*) stands for double knock-out embryos and *++++/−−* stands for overexpression of Mgn cDNA in *Mash1* ^*−/−*^ genetic background embryos. Abbreviations: f, floor plate; r, roof plate; Dap, dorsal alar plate; Lap, lateral alar plate; VLap, ventrolateral alar plate; Lbp, lateral basal plate; Ibp, intermediate basal plate; Mbp, medial basal plate.”

An example that best supports our hypothesis can be seen in the phenotypic analysis of double heterozygous and compound mice, as well as the gain-of-function experiments. In these experiments, *Mgn* was overexpressed in the dMB in the absence of *Mash1* (*Mash1* ^*−/−*^ mice) at levels comparable to those of endogenous *Mgn* expression in the vlMB. Higher levels of MGN homodimers under these conditions were able to trigger the expression of GABAergic markers (*Gad65/67*) in the dMB despite the absence of MGN/MASH1 heterodimers. Thus, high levels of MGN protein in the dorsal aspect overcome the lower efficiency of homodimers and mimic the *in vivo* vlMB conditions of *Mash1* ^*−*/*−*^ mutants where MGN homodimers are sufficient to induce GABAergic fate (neither MGN/MASH1 heterodimers nor MASH1 homodimers can be formed in *Mash1* ^*−*/*−*^ mice). Together, these data indicate a novel mechanistic action in the dorsal/ventrolateral areas governing the induction of MB GABAn based on the differential expression of *Mgn* and *Mash1* at this stage (Fig 7A). Later, at E14.5–E15.5, a conspicuous subpopulation of GABAn was detected in the vmMB in the *Mgn* ^*−*/*−*^ *Mash1* ^*−*/*−*^ double mutants (domains m6–m7). An explanation for this is that this subpopulation of GABAn associated with dopaminergic nuclei (substantia nigra pars reticulata (SNpr) and ventral tegmental area (VTA)) has its developmental origin outside the MB. These neurons migrate out of the ventral rhombomere 1 neuroepithelium in the HB [20], where the GABAergic fate is independent of *Mgn* (*Mgn* is not expressed in the HB during development; Fig 5B).

Taken together, the present data provide compelling evidence that the MB has adopted two crucial factors (MGN and MASH1) and three mechanisms for the acquisition of MB-GABAn during the three distinct phases of development, which are separated in time and place as follows. The earliest wave of GABAergic neurogenesis corresponds to the highest expression level of *Mgn* and *Mash1* mRNA in the vlGABAn at E10.5 and occurs indistinctly in the presence of *Mgn* and/or *Mash1* in a dose-dependent manner. At this stage, MGN or MASH1 homodimers are sufficient to induce GABAn, and heterodimers are not required. The second phase occurs 2 days later (E12.5) in the dMB, where the levels of *Mgn* and *Mash1* expression are lower than in the ventrolateral, which makes the formation of MGN/MASH1 heterodimers a prerequisite for induction, with neither homodimer being sufficient alone. The third population corresponds to GABAn of the SN and VTA and appears at E14.5 in the medial basal/floor plate (domains m6–m7, the vmMB). The fact that none of the *Mgn*/*Mash1* single, double, or compound mutant embryos show a GABAergic phenotype in the SN/VTA indicates that neither MGN/MASH1 heterodimers nor homodimers are necessary for their specification or migration. This explains why *Mgn*, a specific marker for MB-GABAergic neurogenesis, is not expressed in the medial basal/floor plate of the MB at any developmental stage [6]. These different mechanisms define a new model for dorsal/ventrolateral GABAergic neurogenesis (Fig 7B).

### No proliferative function is attributed to *Mgn* or *Mash1* in the vlMB

A role of *Mgn* and *Mash1* in generating the proper number of GABAergic precursor cells in the vlMB cannot be excluded. Given that *Mgn* and *Mash1* can take over each other’s function in d/vMB-GABAergic specification, redundancy may also mask a proliferative function. Extensive analyses performed at the time of GABAergic neurogenesis and later in development indicate that the MB neuronal cytoarchitecture of double mutant mice appears normal despite GABAn constituting a major neuronal population in this region. Several lines of research indicate that *Mgn* ^*−/−*^ *Mash* ^*−/−*^ neurons are generated at proper numbers in VZ of d/vlMB during the first two waves of GABAergic neurogenesis and that the progenitors that should become GABAn do not enter into mutant-specific apoptotic programs but migrate toward the mantle zone. The neuronal citoarchitecture and the molecular analysis of double mutants show that defective neurons are not arrested into a progenitor-like state, since neuronal density of differentiated neurons and expression markers for mature neurons appear to be normal among genotypes. Although some defective cells express glutamatergic markers at the time of GABAergic neurogenesis (Figure B in S2 Fig), it is not clear whether this cells acquire a full glutamatergic identity. Electrophysiological studies are needed to determine whether there is a complete switch of neurotransmitter identity or just a transient acquisition of some glutamatergic markers like *Vglut2*, due to misregulation of GABAergic neurogenesis. Defective GABAn persist during development in double mutant mice and continue to be maintained without expressing GABA until birth. Therefore, massive depletion of d/vlMB-GABAn occurs in *Mgn* ^*−/−*^ *Mash* ^*−/−*^ mutants at the onset of neurogenesis through failure to achieve GABAergic identity rather than deficits in the number of precursor cells or migration cues.

### Downstream GABAergic markers follow the dorsal/ventrolateral context mechanisms

Because all MGN^+^ cells co-express MASH1 in VZ, thereby sharing the pool of presumptive GABAergic precursor cells, it is considered that the high levels of *Mgn* and *Mash1* expression in the ventrolateral domain most likely reflects the limited efficiency of their homodimers to trigger GABAergic neurogenesis rather than an inherent functional difference between heterodimers and homodimers. Consistent with this, the genetic network behind MGN/MASH1 heterodimers and homodimers is essentially maintained along the d/vlMB axis, because both appear to activate the same downstream genetic cascade for neuronal differentiation. Upon ablation of *Mgn* and *Mash1* expression, defective postmitotic GABAn downregulate the expression of transcription factors that are predictive of their GABAergic commitment (*Gad65*, *Gad67*, *Gata2*, *Gata3*, *and Tal1*) and appear to follow the GABA phenotype and the dorsal/ventrolateral context mechanisms proposed above. The *Tal2* gene, expressed in MB-VZ and involved in the GABA pathway, as shown by the lack of GABAn in the *Tal2* ^*−/−*^ mutant [19], appears to function regardless of *Mgn* and *Mash1* genes in the m1-m4 domains. This is particularly evident in the MB of *Mgn* ^*−/−*^ *Mash1* ^*−/−*^ mice, where *Tal2* is expressed without any detectable downregulation, suggesting an independent pathway. Conversely, *Tal2* expression depends on MGN/MASH1 heterodimer in the m5 domain, but not on their homodimers. The m5-GAD^+^ phenotype observed in the *Mgn* ^*−/−*^ *Mash1* ^*−/−*^ mice resembles that of *Tal2* ^*−/−*^ mutants, where few cells in the m5 domain express GAD markers [19].

In summary, we have provided molecular, genetic and phenotypic evidence about two cooperating bHLH factors in the MB for the acquisition of GABAergic identity. We showed distinct mechanisms by which MGN and MASH1 trigger the GABAergic cell fate, which suggest a model for d/vlMB-GABAergic neurogenesis during mouse development. Transcription factors belonging to the hairy/E(spl) and proneural families have long been believed to counterpart each other’s function. This work uncovers a synergistic cooperation between these two families, outlines the underlying different dorsal/ventrolateral mechanisms, and provides a novel paradigm for how they cooperate for the acquisition of MB GABAergic neuronal identity. Finally, an important consideration in MB GABAergic neurogenesis is that these results can help our understanding of the molecular mechanisms underlying neurogenesis in other brain areas and should allow us to understand the functional diversity of these neurons more clearly. Thus, they may facilitate the development of novel insights into the molecular etiologies underlying neurological and psychiatric disorders in which the GABAergic system is disturbed.

## Supporting Information

S1 Material and Methods Oligonucleotides and ISH probes. (DOCX)

S1 Table Summary of yeast-2-hybrid results. (DOCX)

S1 Fig General Y2H screen strategy. Schematic representation for the high-throughput screen using the Y2H assay to detect MGN interactors. The putative interaction between MGN and prey protein reconstituted the function of the Gal4 protein and resulted in *Ade2*, *His3*, and *lacZ* expression, allowing the growth of yeast cells in the synthetic dropout medium (Trp^−^, Leu^−^, His^−^, and Ade^−^). Use of different selection genes (*His3*, *Leu*, *His*, and *Ade*) combined with the *lacZ* reporter gene expressed by different promoters led to the elimination of many false candidates. To assay the specificity of the Y2H interactions, additional transformations were conducted by mating assays (Y2P) **(Figure A)**. Control experiments to assess the quality of the cDNA library. PCR with *Mgn* intron-spanning primers using the cDNA library as a template (line 1), mouse genomic DNA (line 2), and positive (line 3) and negative (line 4) controls; PCR with *Mash1* primers using the cDNA library as a template (line 5) and positive (line 6) and negative (line 7) controls; PCR with *Ngn1* primers using the cDNA library as a template (line 8) and positive (line 9) and negative (line 10) controls **(Figure B)**. To verify that the MGN-fusion protein (pGBKT7-MGN) was expressed in yeast (49 kDa; line 11) compared with the vector alone (24 kDa; line 12), we performed western blot analysis of soluble protein yeast extracts using anti-GAL4-BD antibodies. Negative controls grew only on the medium supplemented with histidine, whereas false-positive clones were negative for *lacZ* staining **(Figure C)**.
(EPS)

S2 Fig Qualitative and quantitative measurements of protein interaction strength. MGN-based pair-wise interaction with proneural factors in 5 mM **(Figure A)** and 15 mM **(Figure B)** of 3-AT as a competitive inhibitor of the yeast His3 protein. Murine p53 fused to the activator domain (AD) and SV40 large T-antigen fused to the binding domain (BD) assay served as the positive interaction control. Single MGN-AD, MGN-BD, MASH1-AD, MASH1-BD, and pGADT7 vectors provided the negative controls. Sensibility assay under increasing concentrations of 3-AT plates. Human Lamin C was used as a negative control **(Figure C)**. Quantification of β-gal activity in yeast cells containing combinations of 2 proteins. The results shown are the average of 5 assays, and the values are expressed as the relative activity of β-gal to the MGN–MGN interaction, which was set arbitrarily to 100% **(Figure D).** The same data was processed as relative values to the p53-large T interaction with SV40 **(Figure E)**. Although the sensitivity of the 5-bromo-4-chloro-3-indolyl-β-d-galactoside (X-gal) assay for weak interactions was higher than that for the O-nitrophenyl β-d-galactopyranoside (ONPG) assay, both approaches ranked the strength of interactions as follows: MGN/MASH1 >> MASH1/NGN2 >> MASH1/MASH1 > MGN/MGN. Error bars represent the standard deviation.
(EPS)

S3 Fig Cells that failed to be GABAn in the double-knockout mice were present in the MB. Immunohistochemical staining of PHH3 in coronal sections. Single cells lining the ventricle are labeled in the vlMBs of wild-type and double-knockout mice (*−−/−−*). No decrease in proliferation was observed in the mutants (PHH3 at E10.5: WT = 84.53±2.44; *−−/−−* = 86.67±3.18; n = 15; p = 0.60. PHH3 at E12.5: WT = 120.70±6.03; *−−/−−* = 124.70±5.15; n = 15; p = 0.62) **(Figure A)**. Immunohistochemical staining of cleaved Caspase 3 around the time of MB GABAergic neurogenesis **(Figure B)** plus TUNEL assays **(Figure C)** were performed to investigate programmed cell death. *Apart from the* typical green autofluorescence from the blood vessels, no increase in apoptosis was observed in the mutants. Apoptosis in the mouse embryo limbs served as the positive control for the cleaved Caspase 3 and TUNEL assays. Scale bars: 25 μm in A, and 100 μm in B and C.
(EPS)

S4 Fig Downregulation of GATA2 and GATA3 factors in double mutants. *In situ* hybridization with the *Gata2* **(Figure A)** and *Gata3* **(Figure B)** riboprobes in sagittal sections (A1, A4, and B1–B4) and in coronal sections (A2–A3, A5–A6 and B5–B9) at E12.5. A strong downregulation of *Gata* expression was observed in the double mutants (*−−/−−*), whereas double heterozygous embryos (*+−/+−*) showed normal vlMB, but with a delay in the induction of dorsal GABAn (A3). The severity of the vlMB GABAergic phenotype also increased in a dose-dependent manner with the number of mutant alleles from the *Mgn* and *Mash1* loci. The phenotypic severity culminated in the absence of GABAn in the double-knockout mice (A6 and B9). Scale bars: 400 μm.
(EPS)

S5 Fig *Lim1* expression depended on MGN/MASH1 heterodimers in the m3 and m4 domains, but was independent of *Mgn* and/or *Mash1* expression in the m5 domain. *In situ* hybridization with the *Lim*1 riboprobe on coronal sections showing the mesencephalon at E12.5. The bars delimit the dorsal/ventral MB subdomains established by Nakatani et al. [10]. Numbers 3, 4, 5, and 6 represents the m3, m4, m5, and m6 domains, respectively. The thick lines indicate the borders of m3-m5 domains, defined by the *Mgn* expression in parallel sections. Scale bar: 400 μm.
(EPS)

S6 Fig *Tal2* expression showed an inverse dependency of *Mgn* and *Mash1* when compared with *Lim1*, whereas *Vglut2* was dependent on *Mgn*, but not on *Mash1*. *In situ* hybridization of E12.5 wild-type, compound, and double-knockout mice showed *Tal2* **(Figure A)** and *Vglut2* **(Figure B)** expression in coronal sections. *Tal2* expression in the m1-m4 domains was not downregulated by neither *Mgn* nor *Mash1*, and its expression in the double knockout mice was comparable with littermate controls. Conversely, expression of *Tal2* in the m5 domain depended on the presence of MGN/MASH1 heterodimers. Upregulation of the *Vglut2* expression depends on *Mgn* (thin arrow), but not on *Mash1* expression, because *Mash1* mutant mice (*++/−−* and *+−/−−*) showed no upregulation of *Vglut2* expression (thick arrow). Scale bars: 500 μm.
(EPS)
