# Supplementary material for: Hairy/Enhancer-of-Split MEGANE and Proneural MASH1 Factors Cooperate Synergistically in Midbrain GABAergic Neurogenesis
Source: PLoS One. 2015 May 20;10(5):e0127681. doi: 10.1371/journal.pone.0127681 (PMC4439124; doi:10.1371/journal.pone.0127681)
Supplement: S1 Material and Methods — (DOCX) [file pone.0127681.s001.docx]

**S1 Material and Methods. Oligonucleotides and ISH probes**

To quantify the levels of mRNAs, RT-qPCR with *Mgn* (Mgn-D: GCATTCCGCGGATTTTCCC and Mgn-R: CCGTAGTGGAAGTAGTTGGC); *Mash1* (Mash1-D: CTCCTGGGAATGGACTTTGGA and Mash1-R: GACGTCGTTGGCGAGAAAC) and the housekeeping genes *ß-actin* (b-actin-D: CAGCTTCTTTGCAGCTCCTTC and b-actin-R: CATGCCGGAGCCGTTGTC), *Gapdh* (Gapdh-D: GTGAAGGTCGGTGTGAACG and Gapdh-R: CGTTGATGGCAACAATCTCC) and *Pgk1* (Pgk1-D: AGCCTCACTGTCCAAACTAGG and Pgk1-R: TCTGTGGCAGATTCACACCC) as controls were used as a forward and reverse oligos, respectively. For the ISH analysis, antisense riboprobes were transcribed from linearized plasmids containing a partial cDNA for *Dbx1* (bp 1117-1944; NM_001005232.1); Gad65 (Gad2) (bp 753-1600; BC018380); Gad67 (Gad1) (bp 934-1786; NM_008077); *Gata2* (bp 229-946; BC107009.2); *Gata3* (bp 388-1196; NM_008091.3); Lim1 (*Lhx1*) (bp 1202-2453; Z27410.1); Mash1 (*Ascl1*) (bp 5-1997; NM_008553.2); Mgn (Helt) (bp 166-1000, DQ294234); Tal2 (bp 332-924; NM_009317.2); Tal1 (bp 921-1472; BC063060.1); *Vglut2* (*Slc17a6*) (bp 1030-1770; NM_080853.2).
