# Supplementary material for: Hairy/Enhancer-of-Split MEGANE and Proneural MASH1 Factors Cooperate Synergistically in Midbrain GABAergic Neurogenesis
Source: PLoS One. 2015 May 20;10(5):e0127681. doi: 10.1371/journal.pone.0127681 (PMC4439124; doi:10.1371/journal.pone.0127681)
Supplement: S1 Table — (DOCX) [file pone.0127681.s002.docx]

S1 Table. Summary of yeast-2-hybrid results.

| **Nr.** | **Locus-ID** | **Gene name** | **Domain description** |
| --- | --- | --- | --- |
| 1 | NM_009697 | Nuclear receptor subfamily 2, group F, member 2 (Nr2f2) | Ligand-binding helical domain-c4 zinc finger |
| 2 | NM_011548 | E2A transcription factor 3 (Tcf3) | Helix-loop-helix DNA-binding domain |
| 3 | NM_011051 | Programmed cell death 6 (Pdcd6) | EF-hand domain; Ca2+-binding protein |
| 4 | NM_010433 | Homeodomain-interacting protein kinase 2 (Hipk2) | Serine/Threonine protein kinases |
| 5 | AK049346 | Megalin/Glycoprotein 330 (Lrp2) | LDL-receptor class A |
| 6 | BC008241 | Proteasome subunit beta type 4 (Psmb4) | Nuclear proteasome beta type-4 subunit. |
| 7 | AK006977 | Adult male testis cDNA Homolog to mitofilin | Mitofilin domain |
| 8 | NM_013904 | Transcription factor HEY-2 (Hey2) | Helix-loop-helix DNA-binding domain; Orange domain |
| 9 | AAH04019 | Mesoderm-specific transcript protein isoform | homoserine O-acetyltransferase |
| 10 | NM_001271444 | NADH dehydrogenase (ubiquinone) Fe-S protein 8 (Ndufs8) | 4Fe-4S double cluster binding domain |
| 11 | NM_009367 | TGFß2 | TGF-beta domain |
| 12 | AK017150 | RIKEN Klon: 5033403L01 |  |
| 13 | AK002874 | RIKEN-Klon: 0610040P02 |  |
| 14 | NM_008553 | Achaete-scute complex homolog 1 Mash1 (Ascl1) | Helix loop helix domain |
| 15 | NM_019299 | Clathrin, heavy polypeptide (Cltc) | Clathrin domain |
| 16 | NM_010419 | Transcription factor HES-5 (Hes5) | Helix-loop-helix domain; Hairy Orange |
| 17 | BC011060 | Nucleoporin 43 (Nup43) | WD40 domain |
| 18 | NM_198326 | NSFL1 (p97) cofactor (p47) (Nsfl1c) | SEP and UBX domains p47-like ubiquitin domain |
| 19 | AF268196 | A dual-specificity phosphatase (VH1) | Ser/Thr and Tyr protein phosphatases |
| 20 | BC013777 | Small nuclear ribonucleoprotein polypeptide A (Snrpa1) | Leucine Rich repeats |
| 21 | NM_153599 | Cyclin-dependent kinase 8 (Cdk8) | Cyclin-dependent protein kinase |
| 22 | DQ294234 | Hairy and Enhancer of split transcription factor Mgn (Helt) | Helix-loop-helix domain; Hairy Orange |
| 23 | NM_009594 | c-abl oncogene 1, non-receptor tyrosine kinase (Abl1) | Src homology SH3 and SH2 domains; mitogen-activated protein kinase domain |
| 24 | NM_011170 | Prion protein | Doppel alpha-helical domain |
| 25 | NM_019567 | Apoptotic chromatin condensation inducer in nucleus (Acin 1) | RRM-ACINU domain |
